# Impact of in-hospital oral beta-blockers initiation on long-term outcomes in ST-elevation myocardial infarction patients with cardiogenic shock

**DOI:** 10.3389/fmed.2025.1666977

**Published:** 2025-10-13

**Authors:** Xiaojin Gao, Mengyuan Liu, Jing Xu, Ling Li, Min Chen, Xinyue Lang, Shaobin Jia, Bin Ning, Haiyan Xu, Lei Song, Yuan Wu, Jun Zhang, Fenghuan Hu, Shubin Qiao, Yongjian Wu, Yanyan Zhao, Yang Wang, Wei Li, Chen Jin, Jingang Yang, Yuejin Yang

**Affiliations:** ^1^Center for Coronary Heart Disease, Department of Cardiology, State Key Laboratory of Cardiovascular Disease, National Center for Cardiovascular Diseases, Chinese Academy of Medical Sciences and Peking Union Medical College, Fuwai Hospital, Beijing, China; ^2^Medical Research and Biometrics Center, State Key Laboratory of Cardiovascular Disease, National Center for Cardiovascular Diseases, Chinese Academy of Medical Sciences and Peking Union Medical College, Fuwai Hospital, Beijing, China; ^3^Department of Cardiology, General Hospital of Ningxia Medical University, Yinchuan, China; ^4^Department of Cardiology, Fuyang People’s Hospital, Fuyang, China

**Keywords:** cardiogenic shock, acute ST-elevation myocardial infarction, beta-blocker, long-term mortality, real-world study

## Abstract

**Background:**

Early revascularization enables ST-elevation myocardial infarction (STEMI) patients with cardiogenic shock (CS) to initiate oral beta-blockers once hemodynamic stability is achieved, but the impact of such initiation on prognosis remains unknown. We aimed to describe the clinical use of oral beta-blockers and assess its impact on long-term outcomes in STEMI patients with CS in a real-world setting.

**Materials and methods:**

The China Acute Myocardial Infarction registry (CAMI) is a prospective observational study that enrolls patients with acute myocardial infarction from three-level hospitals across 31 administrative regions in mainland China. Among 19,112 STEMI patients in the CAMI registry, a total of 744 STEMI patients who presented with CS at admission were analyzed. Multivariate regression models were used to evaluate the impact of in-hospital oral beta-blockers on 2-year outcomes. Inverse probability treatment weighting (IPTW) score was further used to address biases between the groups with and without oral beta-blockers. The primary endpoint was all-cause death.

**Results:**

42.7% (*n* = 318) of the patients initiated in-hospital oral beta-blockers; these patients were in better states and more likely to receive primary percutaneous coronary intervention and secondary prevention at discharge. The crude 2-year all-cause mortality was 41.7%, with a lower rate in patients who received oral beta-blockers (24.2% vs. 54.8%, *P* < 0.001). However, after multivariate adjustment, patients who received oral beta-blockers showed a non-significant increase in 2-year mortality compared with non-users (HR = 1.29, 95% CI: 0.95–1.75, *P* = 0.099), and this increase became statistically significant in the subgroup of county-level hospitals (HR = 1.79, 95% CI: 1.03–3.09, *P* = 0.038, *P*-interaction = 0.010). Furthermore, after balancing the baseline covariates using IPTW and further adjusting for discharge medications, initiation of oral beta-blockers during hospitalization increased the risk of 2-year all-cause mortality (HR = 1.59, 95% CI: 1.18–2.13, *P* = 0.002).

**Conclusion:**

No benefit of in-hospital oral beta-blockers initiation on long-term all-cause mortality was found in Chinese STEMI patients with CS, and a trend toward increased mortality existed, especially in small-scale hospitals with insufficient experience in CS treatment.

## Graphical Abstract

Patients without oral beta-blockers were treated as the reference group when calculating HR. 
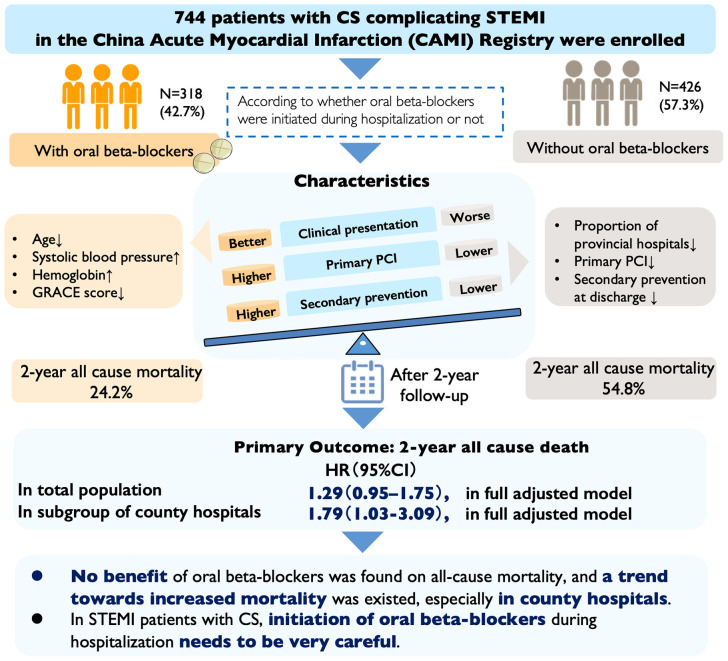

## 1 Introduction

Cardiogenic shock (CS) is defined as a state of ineffective cardiac output, which causes a series of clinical and biochemical manifestations of inadequate end-organ perfusion (1, 2). More than 80% of CS cases are infarct-related, resulting from ventricular failure and mechanical complications subsequent to acute myocardial infarction (AMI) (3). Despite the popularization of early revascularization and advancement in the care for AMI patients, CS remains the most common cause of death in AMI (4, 5). In ST-elevation myocardial infarction (STEMI) cases, nearly 6–10% of them are complicated by CS, with in-hospital mortality higher than 50% (6). These patients are also more likely to suffer from out-of-hospital cardiac arrest and have relevant morbidity and mortality higher than 50% after one year (7–9). Therefore, optimizing management is critical to reduce mortality and improve prognosis in patients with STEMI complicated by CS.

Beta-blockers were considered a fundamental component of medication in patients with AMI (10–13). Due to their negative chronotropic and inotropic effects, beta-blockers could decrease myocardial oxygen consumption, thereby improving myocardial oxygenation during AMI (14). The use of beta-blockers was also found to increase the threshold of ventricular tachycardia and limit the adverse effects of sympathetic activity on cardiac regeneration, thus preventing sudden cardiac death and improving the prognosis of AMI (14–17). Therefore, in hemodynamically stable patients with STEMI, early use of oral beta-blockers should be considered within the first 24 h according to current guidelines (10, 12, 13). Continuation of beta-blockers was also recommended during and after hospitalization for all STEMI patients without contraindications (10, 12).

However, beta-blockers are contraindicated in patients with CS in current guidelines due to their negative inotropic effects (13, 18). Thus, how to balance their cardiovascular protective effects and their negative effects on hemodynamics merits further study among patients with infarct-related CS. Unfortunately, as it is not practical to conduct clinical trials on such critically ill patients, whether initiating oral beta-blocker during hospitalization is beneficial to STEMI patients with CS is inconclusive. To date, only a few studies have investigated the effect of beta-blockers on the prognosis of patients with CS, and these studies have mainly focused on the prehospital setting (19, 20). Furthermore, there is a lack of real-world studies on the current use and characteristics of beta-blockers in STEMI patients with CS. Therefore, in the present study, we aim to describe the clinical use of oral beta-blockers during hospitalization and its impact on long-term outcomes in Chinese patients with CS complicating STEMI in a real-world setting.

## 2 Materials and methods

### 2.1 Study population

**FIGURE 1:**
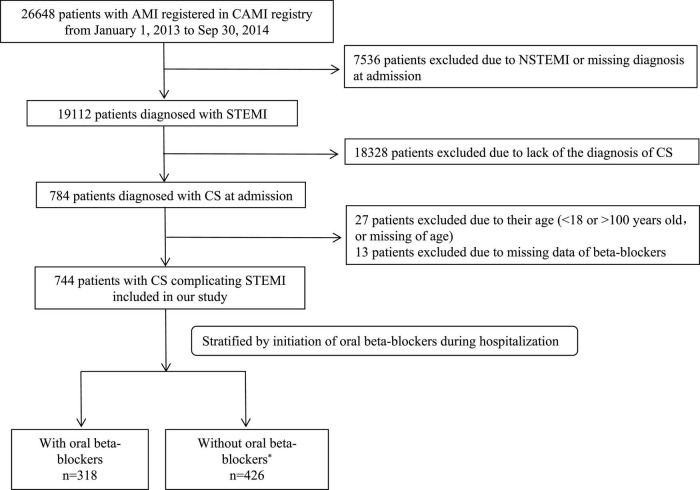
Flow chart of the procedure of the study. *At discharge, if a surviving patient was not discharged with oral beta-blocker but had used it during hospitalization, he/she was treated as intolerance oral beta-blockers and was included in group without oral beta-blockers (*n* = 52).

The China Acute Myocardial Infarction (CAMI) Registry is a prospective, nationwide, multicenter observational study for Chinese patients with AMI (NCT01874691). The detailed procedures of this registry have been described previously (21). In brief, the registry included 108 hospitals across three tiers (provincial-level, prefectural-level and county-level hospitals) from 27 provinces and four municipalities in Mainland China, which sufficiently reflects hospital practices nationwide (21). Provincial hospitals are defined as academic hospitals affiliated to universities located in provincial capitals. Prefecture-level hospitals and county-level hospitals are located in medium-sized cities and in the smallest cities, respectively (22). Initially, a total of 19,112 patients with STEMI were enrolled between 1 January 2013 and 30 September 2014. Among these patients, 4.1% (*n* = 784) were diagnosed with CS upon admission and included in the analysis. CS was defined as either Killip Class IV or a clinical diagnosis of CS by experienced clinicians. The specific criteria included systolic blood pressure < 90 mmHg more than 30 min or the need for catecholamines to maintain systolic pressure > 90 mmHg; clinical pulmonary congestion; and impaired end-organ perfusion manifested by at least one of the following: altered mental status, cold and clammy skin and extremities, oliguria with a urine output < 30 mL/h, or an arterial lactate level > 2.0 mmol/L (4, 8, 23). After excluding patients aged > 100 years or < 18 years, those with missing age data, and those without complete data for in-hospital initiation of oral beta-blockers, 744 eligible patients were finally included in the analysis (Figure 1). Patients were further divided into two groups based on whether oral beta-blockers were initiated during hospitalization: the beta-blocker group and the non-beta-blocker group. Specifically, patients discharged on oral beta-blockers were included in the former group, while the latter group included surviving patients who were not discharged with oral beta-blockers but did use them during hospitalization. Oral beta-blockers were carefully prescribed when the patients’ hemodynamics were relatively stable, and this decision was made by experienced clinicians after weighing the pros and cons. This study was approved by the ethics committee of Fuwai Hospital and by the ethics committees of each participating institution. Written informed consent was obtained from each patient.

### 2.2 Data collection

In the CAMI registry, patient data at each participating site was entered into computers using a fixed electronic case report form, including demographic characteristics, medical history, clinical presentation, in-hospital treatments, discharge medications and outcomes. This collected data was further validated and submitted via a secure, web-based electronic data capture system by locally trained investigators. Follow-up visits are planned at 30 days, 6, 12, 18 and 24 months. The events were reviewed and collected either in person during clinic visits or via telephone calls. Clinical events must be validated by source documents (21). A series of standardized measures have been implemented to ensure data quality in the registry (21, 24).

### 2.3 Outcome definitions

The primary outcome was 2-year all-cause death. The secondary outcomes included in-hospital, 30-day, 1-year all-cause death, as well as in-hospital, 30-day, 1-year, 2-year major adverse cardiovascular and cerebrovascular events (MACCE), recurrent MI, stroke, revascularization, and TIMI major bleeding. MACCE was defined as a composite of all-cause death, recurrent MI, and stroke. Recurrent MI was diagnosed if the patient met at least two of the following criteria: unrelieved onset of chest pain lasting for 20 min; elevation of myocardial enzymes (troponin T, troponin I, or creatine kinase-MB) ≥ three times the upper limit of normal; and new changes in the ST-segment or Q waves on electrocardiograms, indicating new myocardial injury. Revascularization was defined as an emergent intervention induced by the ischemia in a previously treated vessel or by new thrombosis. TIMI major bleeding was diagnosed if there was a reduction in hemoglobin ≥ 5 g/L (or > 15% in hematocrit) or any intracranial bleeding (25).

### 2.4 Statistical analysis

Normally distributed continuous variables are expressed as mean ± standard deviation (SD), and non-normally distributed variables are expressed as median (interquartile range, IQR). Categorical variables are shown as number (percentage). The differences among patients classified according to the initiation of beta-blockers during hospitalization were compared using the Student’s *t*-test for normally distributed continuous variables. Categorical variables were compared using the χ^2^ test or Fisher’s exact test. For non-normally distributed data, the Wilcoxon rank test was used. Univariate and multivariate COX regression models were built to evaluate the effect of initiation of oral beta-blockers during hospitalization on in-hospital, 30-day, 1-year, and 2-year outcomes. Adjusted variables were either statistically significant in univariate analysis or clinically critical, including age (≥ 75 years or not), gender, history of diabetes, hypertension, or heart failure, heart rate (> 100 beats/min or not), anterior MI, primary percutaneous coronary intervention (PCI), hospital level, and discharge medications including aspirin, P2Y12 receptor inhibitor, and statin (discharge medications were not adjusted when considering in-hospital outcomes). These results were reported as hazard ratios (HRs) with 95% confidence intervals (95% CIs). Prior to modeling, multicollinearity among the variables was assessed using the variance inflation factor (Supplementary Table 1). Kaplan–Meier survival curves and the log-rank test were used to test for differences in survival status among groups with or without oral beta-blockers. Subgroup and interaction analyses were performed according to age, gender, level of hospital, heart rate, left ventricular ejection fraction (LVEF), anterior MI, and primary PCI. Landmark analysis was performed for primary outcome by dividing the entire 2-year follow-up period into the first 30 days and days 31 to 2 years. To address biases between the exposed and unexposed groups due to lack of randomization, the inverse probability treatment weighting (IPTW) score was also used. The propensity score of being in the beta-blockers group was estimated by a logistic regression model, which included the following covariates: age (≥ 75 years or not), gender, current smoking, history of diabetes, hypertension, or heart failure, heart rate (> 100 beats/min or not), anterior MI, primary PCI, and hospital level as categorical variables; and GRACE score, systolic blood pressure (SBP) on admission as continuous variables. Covariates considered to have influence on initiation of oral beta-blockers or have prognostic significance were selected. IPTW was calculated through assigning the inverse of the probability for the beta-blockers-exposed group and the inverse of one minus the probability for the unexposed group. The standardized mean differences (SMD) of the baseline covariates between the two groups before and after IPTW were presented in Supplementary Table 2, and an SMD ≤ 0.1 was considered a good balance between two groups (26, 27). To study the effect of oral beta-blockers on primary outcome, a Cox regression model was used for 2-year all-cause death, and discharge medications including aspirin, P2Y12 receptor inhibitor, and statin were further adjusted. All analyses were two-sided, and statistical significance was defined as *P* < 0.05. Statistical analysis was performed using SAS software version 9.4 (SAS Institute Inc., Cary, NC, USA).

## 3 Results

### 3.1 Baseline patient characteristics

The characteristics of patients, both overall and stratified by initiation of beta-blockers during hospitalization, are shown in Table 1. A total of 744 participants with an average age of 65.1 ± 12.3 years were included in the analysis, and 70.6% (*n* = 525) of them were men. Current smoking was present in 39.0% (*n* = 289) of participants, hypertension in 47.3% (*n* = 339), diabetes in 18.7% (*n* = 128), hyperlipidemia in 6.0% (*n* = 35), and prior MI in 6.7% (*n* = 46). Oral beta-blocker therapy was initiated in 318 patients, accounting for 42.7% of the population with CS complicating STEMI. Patients with oral beta-blockers presented with a younger age (62.7 ± 12.0 vs. 67.0 ± 12.2), a higher proportion of male patients (78.9% vs. 64.3%), a higher rate of cigarette use (44.7% vs. 34.8%), a higher level of SBP (95.9 ± 27.5 vs. 89.4 ± 27.9) and hemoglobin (134.7 ± 20.4 vs. 131.0 ± 24.6), a higher percentage of primary PCI (42.5% vs. 30.5%), a lower GRACE score (159.7 ± 30.5 vs. 168.2 ± 32.5) and a lower percentage of vasoactive agents use (23.0% vs. 30.3%). Initiation of oral beta-blockers seemed to be more frequent in provincial hospitals (29.2% vs. 20.7%). Besides, patients who received oral beta-blocker therapy had a significantly lower rate of transfer to a higher-level hospital (3.8% vs. 10.8%); consequently, they had a longer length of stay [12.0 (8.0–16.0) vs. 5.0 (1.0–12.0)], whether in the intensive care unit or in the general ward. However, no significant differences were observed in the mean values of body mass index, heart rate, LVEF and serum creatinine between the two groups. Additionally, the proportions of patients with a previous history of cardiovascular disease, anterior or right ventricular MI, or who received thrombolytic therapy or emergency coronary artery bypass grafting did not differ significantly between the two groups. No significant difference was found between the two groups regarding the proportion of adjunctive treatments.

**TABLE 1:** Baseline characteristics and treatment during hospitalization of study population stratified by initiation of oral beta-blockers.

Variable	Total (*n* = 744)	With beta-blockers (*n* = 318)	Without beta-blockers (*n* = 426)	*P*-value
Age, mean ± SD, years	65.1 ± 12.3	62.7 ± 12.0	67.0 ± 12.2	< 0.001
Age ≥ 75 years, *n* (%)	181 (24.3)	52 (16.4)	129 (30.3)	< 0.001
Male, *n* (%)	525 (70.6)	251 (78.9)	274 (64.3)	< 0.001
BMI, mean ± SD, kg/m^2^	23.9 ± 6.1	24.1 ± 8.6	23.7 ± 2.9	0.526
Current smoking, *n* (%)	289 (39.0)	142 (44.7)	147 (34.8)	0.006
Level of hospital, *n* (%)				
Provincial	181 (24.3)	93 (29.2)	88 (20.7)	0.003
Prefectural	400 (53.8)	171 (53.8)	229 (53.8)	
County	163 (21.9)	54 (17.0)	109 (25.6)	
**History of disease**				
Hypertension, *n* (%)	339 (47.3)	142 (45.5)	197 (48.8)	0.388
Diabetes, *n* (%)	128 (18.7)	47 (15.7)	81 (21.0)	0.074
Hyperlipidemia, *n* (%)	35 (6.0)	20 (7.6)	15 (4.7)	0.139
Prior MI, *n* (%)	46 (6.7)	17 (5.7)	29 (7.5)	0.330
Prior PCI, *n* (%)	36 (5.0)	15 (4.9)	21 (5.1)	0.882
Prior CABG, *n* (%)*	1 (0.1)	0 (0.0)	1 (0.2)	1.000
Prior HF, *n* (%)	21 (3.0)	5 (1.6)	16 (4.0)	0.060
Prior non-hemorrhagic stroke, *n* (%)	69 (9.5)	25 (8.0)	44 (10.7)	0.209
**At presentation**				
** Physical examination**				
Heart rate, median (IQR), beats/min	75.0 (55.0, 98.0)	75.0 (56.0, 96.0)	75.0 (54.0, 100.0)	0.691
Heart rate > 100 beats/min, *n* (%)	162 (21.8)	61 (19.2)	101 (23.7)	0.137
SBP, mean ± SD, mmHg	92.2 ± 27.9	95.9 ± 27.5	89.4 ± 27.9	0.002
SBP < 90 mmHg, *n* (%)	401 (53.9)	154 (48.4)	247 (58.0)	0.010
** Other characteristics**				
GRACE Score, mean ± SD	164.6 ± 31.9	159.7 ± 30.5	168.2 ± 32.5	< 0.001
Anterior MI, *n* (%)	334 (45.0)	151 (47.8)	183 (43.0)	0.191
Right ventricular MI, *n* (%)	155 (20.9)	62 (19.6)	93 (21.8)	0.463
Use of vasoactive agents, *n* (%)	202 (27.2)	73 (23.0)	129 (30.3)	0.025
Cardiac arrest, *n* (%)	108 (14.5)	44 (13.9)	64 (15.0)	0.661
Cardiopulmonary resuscitation, *n* (%)	73 (24.7)	34 (29.6)	39 (21.7)	0.128
Pre-hospital delay, *n* (%)				0.771
< 3 h	232 (31.5)	98 (31.2)	134 (31.7)	
3–6 h	205 (27.8)	91 (29.0)	114 (27.0)	
6–12 h	95 (12.9)	43 (13.7)	52 (12.3)	
≥ 12 h	205 (27.8)	82 (26.1)	123 (29.1)	
**Laboratory and imaging findings on admission**				
LVEF,%	50.5 ± 12.4	51.0 ± 11.8	49.9 ± 13.1	0.382
LVEF < 50%, *n* (%)	183 (41.3)	101 (41.6)	82 (41.0)	0.905
Hemoglobin, mean ± SD, g/L	132.6 ± 22.9	134.7 ± 20.4	131.0 ± 24.6	0.030
Scr, median (IQR), mg/dL	92.1 (73.0, 124.8)	89.0 (71.6, 113.0)	95.7 (74.0, 130.0)	0.764
**Treatment strategy**				
Primary PCI, *n* (%)	265 (35.6)	135 (42.5)	130 (30.5)	< 0.001
Thrombolysis, *n* (%)	117 (15.7)	52 (16.4)	65 (15.3)	0.686
Emergency CABG, *n* (%)*	3 (0.4)	2 (0.6)	1 (0.2)	0.579
**Adjunctive therapy during hospitalization**				
Temporary pacemaker, *n* (%)	64 (8.6)	24 (7.5)	40 (9.4)	0.373
IABP, *n* (%)	100 (13.6)	40 (12.7)	60 (14.2)	0.539
**Length of stay**				
Average length of stay, median (IQR), day	9.0 (2.0–15.0)	12.0 (8.0–16.0)	5.0 (1.0–12.0)	< 0.001
Inthe intensive care unit, median (IQR), day	2.0 (0.5–7.0)	4.0 (1.0–8.0)	1.0 (0.0–5.0)	< 0.001
Inthe general ward, median (IQR), day	3.0 (0.0–9.0)	6.0 (1.0–11.0)	1.0 (0.0–7.0)	< 0.001
**Transfer to superior hospital, *n* (%)**	44 (7.4%)	11 (3.8%)	33 (10.8%)	< 0.001

BMI, body mass index; CABG, coronary artery bypass graft; HF, heart failure; IABP, intra-aortic balloon pump; IQR, interquartile range; LVEF, left ventricular ejection fraction; MI, myocardial infarction; PCI, percutaneous coronary intervention; SBP, systolic blood pressure; Scr, serum creatinine; SD, standard deviation.

*Fisher’s exact test was used.

### 3.2 Discharge medications

As for medications at discharge, secondary prevention drugs such as aspirin, P2Y12 receptor inhibitors, statins were used more frequently at discharge in the group receiving oral beta-blocker therapy (Supplementary Table 3). Other drugs, including angiotensin-converting enzyme inhibitors/angiotensin receptor blockers (ACEI/ARBs) or nitrates were also administered more often as discharge medications in the group receiving oral beta-blockers. However, the proportions of patients receiving calcium antagonists, mineral-corticoid receptor antagonists and diuretic therapy were similar between the two groups.

### 3.3 In-hospital outcomes and complications

As for in-hospital outcomes, the incidence of all-cause death was 33.2% in the total study population, and it was significantly lower in patients who received oral beta-blocker therapy than in those without (16.7% vs. 45.5%, *P* < 0.001) (Table 2). Similarly, the incidence of MACCE in patients with oral beta-blocker therapy was significantly lower than that in patients without beta-blocker therapy (19.2% vs. 46.9%, *P* < 0.001). The rates of recurrent MI, stoke and TIMI major bleeding were all similar between the two groups (*P* > 0.05) (Table 2). Compared with patients who did not initiate beta-blockers, there was a significantly lower rate of cardiac arrest, ventricular tachycardia or ventricular flutter, atrial flutter or atrial fibrillation, and sinus arrest or severe bradycardia during hospitalization in patients who initiated oral beta-blockers.

**TABLE 2:** In-hospital outcome and complications of study population stratified by initiation of oral beta-blockers.

Variable	Total (*n* = 744)	With beta-blockers (*n* = 318)	Without beta-blockers (*n* = 426)	*P*-value
**In-hospital outcome**				
All-cause death, *n* (%)	247 (33.2)	53 (16.7)	194 (45.5)	< 0.001
MACCE*, *n* (%)	261 (35.1)	61 (19.2)	200 (46.9)	< 0.001
Recurrent MI, *n* (%)	12 (1.6)	5 (1.6)	7 (1.6)	0.939
Stroke, *n* (%)	16 (2.2)	7 (2.2)	9 (2.1)	0.934
TIMI major bleeding, *n* (%)^†^	7 (0.9)	2 (0.6)	5 (1.2)	0.705
**Complications during hospitalization**				
Mechanical complications, *n* (%)	18 (2.4)	4 (1.3)	14 (3.3)	0.065
Cardiac arrest, *n* (%)	174 (23.5)	37 (11.7)	137 (32.3)	< 0.001
VT/VF, *n* (%)	164 (22.1)	59 (18.6)	105 (24.8)	0.045
Atrial flutter/atrial fibrillation, *n* (%)	50 (6.7)	14 (4.4)	36 (8.5)	0.025
Sinus arrest/severe bradycardia, *n* (%)	49 (6.6)	9 (2.8)	40 (9.4)	< 0.001
Second degree AVB or above, *n* (%)	89 (12.0)	31 (9.8)	58 (13.7)	0.103

*MACCE represents a composite of all-cause death, recurrent MI and stroke. AVB, atrioventricular block; MACCE, major adverse cardiovascular and cerebrovascular event; MI, myocardial infarction; VF, ventricular flutter; VT, ventricular tachycardia.

^†^ Fisher’s exact test was used.

The results of the univariate and multivariate Cox regression analysis assessing the impact of initiation of oral beta-blockers on in-hospital outcomes were presented in Supplementary Table 4. Initiation of oral beta-blockers was associated with a lower risk of in-hospital all-cause mortality after adjustment for age, gender, history of diabetes, hypertension or heart failure, heart rate, anterior MI, primary PCI and hospital level (HR = 0.37, 95% CI: 0.27–0.50, *P* < 0.001). Besides, initiation of oral beta-blockers was independently associated with a lower risk of in-hospital MACCE (HR = 0.40, 95% CI: 0.29–0.53, *P* < 0.001).

### 3.4 Outcomes at 30-day, 1-year and 2-year follow-up

The follow-up rate was 99.6, 98.4 and 97.6% at 30-day, 1-year and 2-year follow-up, respectively. The 30-day, 1-year and 2-year all-cause mortality were 35.0, 39.8 and 41.7%, respectively, in the total study population (Table 3). Compared with patients without oral beta-blockers, patients with oral beta-blockers had a significantly lower 30-day, 1-year and 2-year all-cause mortality (17.7% vs. 47.9%, 22.1% vs. 52.9% and 24.2% vs. 54.8%, *P* < 0.001). Besides, the 30-day, 1-year and 2-year incidences of MACCE were all lower in patients with oral beta-blocker therapy than those in patients without (*P* < 0.001) (Table 3).

**TABLE 3:** Outcome of study population at follow-up, stratified by initiation of oral beta-blockers.

Variable	Total (*n* = 744)	With beta-blockers (*n* = 318)	Without beta-blockers (*n* = 426)	*P*-value
**30-day follow-up**, *n*	741	317	424	
All-cause death, *n* (%)	259 (35.0)	56 (17.7)	203 (47.9)	< 0.001
MACCE*, *n* (%)	272 (36.7)	64 (20.2)	208 (49.1)	< 0.001
Recurrent MI, *n* (%)	13 (1.8)	5 (1.6)	8 (1.9)	0.750
Stroke, *n* (%)	17 (2.3)	8 (2.5)	9 (2.1)	0.719
Revascularization, *n* (%)	26 (3.5)	12 (3.8)	14 (3.3)	0.724
TIMI major bleeding, *n* (%)^†^	7 (0.9)	2 (0.6)	5 (1.2)	0.705
**1-year follow-up**, *n*	732	312	420	
All-cause death, *n* (%)	291 (39.8)	69 (22.1)	222 (52.9)	< 0.001
MACCE*, *n* (%)	308 (42.0)	79 (25.3)	229 (54.4)	< 0.001
Recurrent MI, *n* (%)	17 (2.3)	8 (2.6)	9 (2.1)	0.709
Stroke, *n* (%)	19 (2.6)	8 (2.6)	11 (2.6)	0.967
Revascularization, *n* (%)	40 (5.5)	20 (6.4)	20 (4.8)	0.331
TIMI major bleeding, *n* (%)^†^	8 (1.1)	3 (1.0)	5 (1.2)	1.000
**2-year follow-up**, *n*	726	310	416	
All-cause death, *n* (%)	303 (41.7)	75 (24.2)	228 (54.8)	< 0.001
MACCE*, *n* (%)	323 (44.4)	85 (27.4)	238 (57.1)	< 0.001
Recurrent MI, *n* (%)	19 (2.6)	8 (2.6)	11 (2.6)	0.958
Stroke, *n* (%)	20 (2.8)	8 (2.6)	12 (2.9)	0.808
Revascularization, *n* (%)	49 (6.7)	22 (7.1)	27 (6.4)	0.728
TIMI major bleeding, *n* (%)^†^	9 (1.2)	4 (1.3)	5 (1.2)	1.000

*MACCE represents a composite of all-cause death, recurrent MI and stroke. MACCE, major adverse cardiovascular and cerebrovascular event; MI, myocardial infarction.

^†^ Fisher’s exact test was used.

**FIGURE 2:**
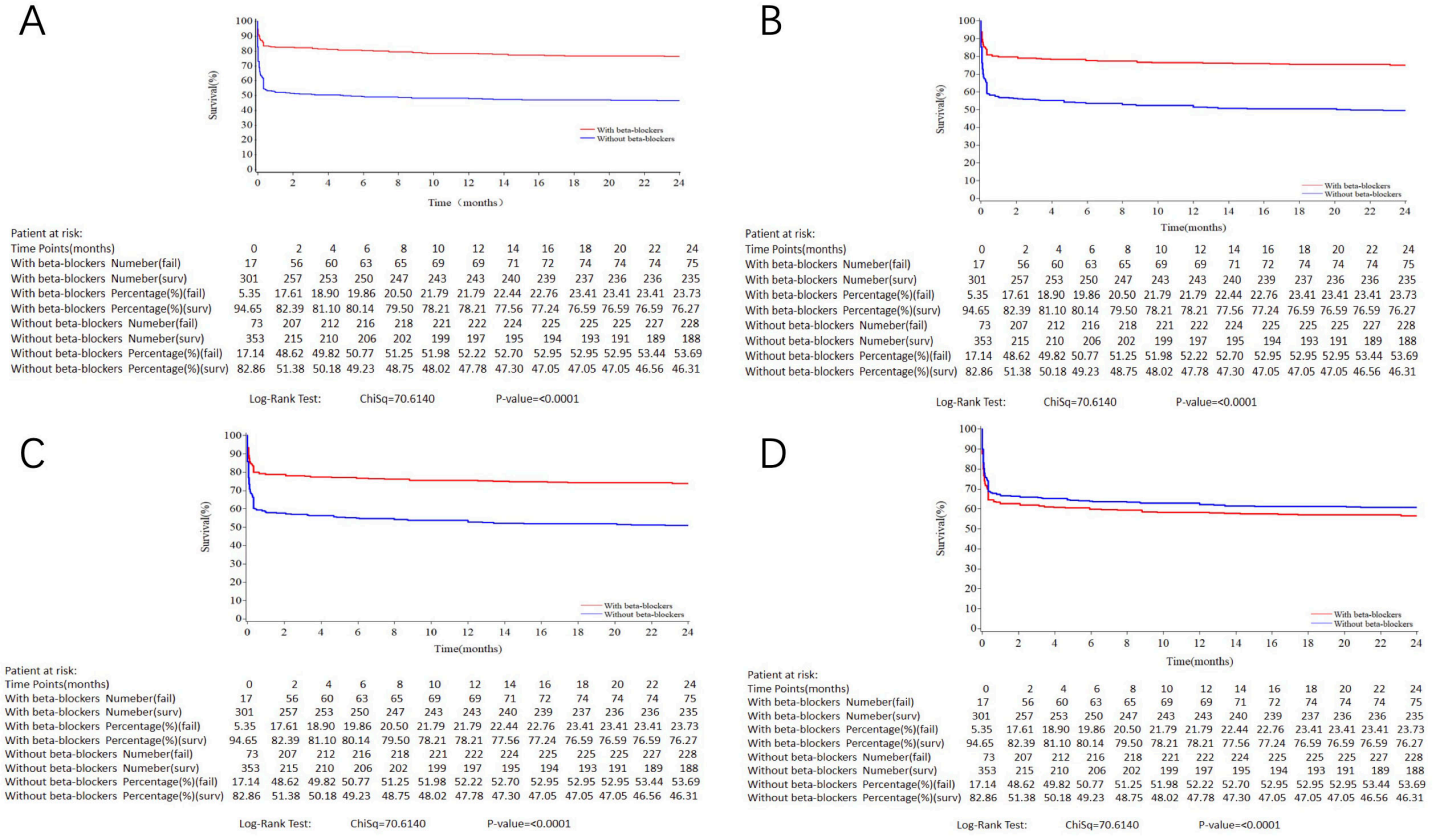
Kaplan–Meier curves for 2-year all-cause death in the study population stratified by initiation of oral beta-blockers. **(A)** crude model. **(B)** Variables included in the adjusted model 1 were initiation of oral beta-blockers, age, gender. **(C)** Variables included in the adjusted model 2 were: initiation of oral beta-blockers, age, gender, primary PCI. **(D)** Variables included in the adjusted model 3 were: initiation of oral beta-blockers, age, gender, history of diabetes, hypertension or heart failure, heart rate, anterior MI, primary PCI, level of hospital and discharge medication including aspirin, P2Y12 receptor inhibitor and statin. Log-rank *P* < 0.001.

Table 4 presents the results of multivariate Cox regression analysis for the effect of oral beta-blockers on 2-year all-cause mortality. Initiation of oral beta-blockers was associated with a lower risk of 2-year all-cause mortality in the crude model, the adjusted model 1 and the adjusted model 2; however, this benefit of oral beta-blockers disappeared and tended to be harmful in the fully adjusted model (HR = 1.29, 95% CI: 0.95–1.75, *P* = 0.099). Kaplan–Meier survival curves reveal that the impact of initiation of oral beta-blockers changed from protective to harmful on 2-year all-cause mortality (Figure 2). As for secondary outcomes, there were significant associations between the initiation of oral beta-blocker and an increased risk of MACCE (HR = 1.39, 95% CI: 1.01–1.90, *P* = 0.045) and recurrent MI (HR = 4.10, 95% CI: 1.02–16.40, *P* = 0.046) at 30 days after adjustment for other covariates. However, initiation of oral beta-blocker was not associated with other secondary outcomes at 30-day, 1-year and 2-year follow-up in the fully adjusted model (Supplementary Table 4). Supplementary Table 5 and Supplementary Figure 1 present the landmark analysis of all-cause death occurring within and after 30 days, and the landmark analysis after 30 days showed no difference between the two groups (HR = 0.98, 95% CI: 0.50–1.93, *P* = 0.958).

**TABLE 4:** Multivariable Cox regression model predicting incidence of 2-year all-cause death.

Variable	Crude model		Adjusted model 1		Adjusted model 2		Adjusted model 3	
	HR (95% CI)	*P*-value	HR (95% CI)	*P*-value	HR (95% CI)	*P*-value	HR (95% CI)	*P*-value
Initiation of oral beta-blockers	0.35 (0.27, 0.46)	< 0.001	0.40 (0.30, 0.52)	< 0.001	0.43 (0.33, 0.56)	< 0.001	1.29 (0.95, 1.75)	0.099
Female	1.88 (1.49, 2.36)	< 0.001	1.47 (1.15, 1.86)	0.002	1.48 (1.16, 1.88)	0.001	1.22 (0.95, 1.56)	0.121
Age ≥ 75 years	2.02 (1.60, 2.56)	< 0.001	1.61 (1.26, 2.06)	< 0.001	1.49 (1.16, 1.91)	0.002	1.34 (1.04, 1.72)	0.024
History of diabetes	1.24 (0.93, 1.64)	0.143	–	–	–	–	1.02 (0.76, 1.36)	0.891
History of hypertension	1.06 (0.85, 1.33)	0.606	–	–	–	–	0.84 (0.66, 1.07)	0.157
History of HF	1.99 (1.16, 3.41)	0.012	–	–	–	–	1.69 (0.98, 2.92)	0.061
Heart rate > 100 beats/min	2.02 (1.58, 2.57)	< 0.001	–	–	–	–	1.19 (0.92, 1.53)	0.186
Anterior MI	1.83 (1.46, 2.30)	< 0.001	–	–	–	–	1.47(1.16, 1.87)	0.001
Primary PCI	0.40 (0.30, 0.52)	< 0.001	–	–	0.46 (0.35, 0.61)	< 0.001	0.70 (0.52, 0.94)	0.017
Level of hospital								
County	Ref.		–	–	–		Ref.	
Prefectural	0.74 (0.57, 0.96)	0.026	–	–	–	–	1.23 (0.94, 1.62)	0.126
Provincial	0.39 (0.27, 0.55)	< 0.001	–	–	–	–	0.62 (0.42, 0.90)	0.012
**Discharge medication**								
Aspirin	0.08 (0.06, 0.11)	< 0.001	–	–	–	–	0.37 (0.19, 0.72)	0.003
P2Y12 receptor inhibitor	0.08 (0.06, 0.11)	< 0.001	–	–	–	–	0.45 (0.24, 0.86)	0.016
Statin	0.08 (0.06, 0.11)	< 0.001	–	–	–	–	0.36 (0.20, 0.64)	< 0.001

Variables included in the adjusted model 1 were: initiation of oral beta-blockers, age, gender. Variables included in the adjusted model 2 were: initiation of oral beta-blockers, age, gender, primary PCI. Variables included in the adjusted model 3 (fully adjusted model) were: initiation of oral beta-blockers, age, gender, history of diabetes, hypertension or HF, heart rate, anterior MI, primary PCI, level of hospital and discharge medication including aspirin, P2Y12 receptor inhibitor and statin. CI, confidence interval; HF, heart failure; MI, myocardial infarction; HR, hazard ratio; PCI, percutaneous coronary intervention.

### 3.5 IPTW

We further used IPTW to address biases between the group with and without oral beta-blockers. Upon adjustment by IPTW, patient characteristics of the two groups were well balanced in terms of the relevant baseline covariates (Supplementary Table 2). Initiation of oral beta-blockers was associated with a lower risk of 2-year all-cause mortality (HR = 0.48, 95% CI: 0.37–0.62, *P* < 0.001). However, after further adjustment for discharge medication, initiation of oral beta-blockers was associated with an increased risk of 2-year all-cause mortality (HR = 1.59, 95% CI: 1.18–2.13, *P* = 0.002).

### 3.6 Subgroup analysis

**FIGURE 3:**
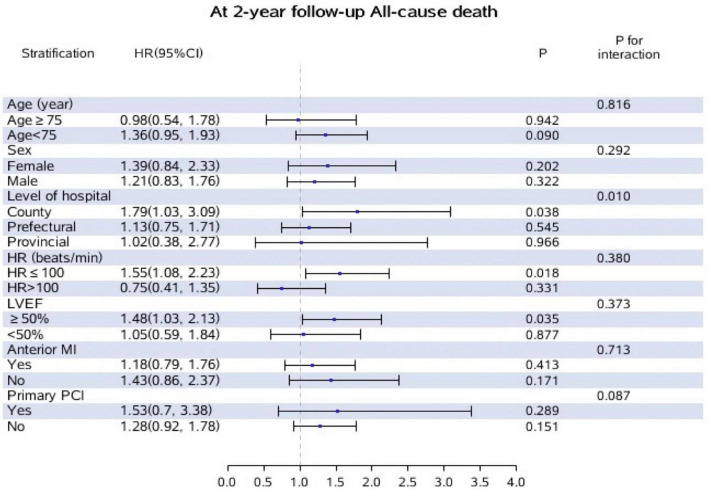
Subgroup and interaction analyses for the effect of oral beta-blockers on incidence of all-cause death at 2-year follow-up according to different variables. Adjusted for, if not stratified by age, gender, level of hospital, heart rate, LVEF, anterior MI, primary PCI. Patients who did not initiated oral beta-blockers during hospitalization were treated as the reference group.

The results of subgroup and interaction analyses in the whole population were shown in Figure 3. There was an apparent interaction between hospital level and initiation of oral beta-blockers with regard to 2-year all-cause mortality (*P*-interaction = 0.010). When stratified by hospital level, the results showed that initiation of oral beta-blockers was significantly associated with higher incidence of 2-year all-cause death in county hospitals (HR = 1.79, 95% CI: 1.03–3.09, *P* = 0.038) but not in prefectural or provincial hospitals. The Kaplan–Meier survival curve and baseline characteristics stratified by three hospital levels are shown in Supplementary Figure 2 and Supplementary Table 6. No interaction was found between the other covariates and initiation of oral beta-blockers in our analysis.

## 4 Discussion

The major findings of the present study were as follows. 42.7% of the STEMI patients with CS initiated oral beta-blocker therapy during hospitalization, who had younger age, higher level of systolic blood pressure and hemoglobin, and a lower GRACE score. Initiation of oral beta-blockers was more likely in provincial hospitals, and these patients were more likely to receive primary PCI and secondary prevention at discharge. The 2-year all-cause mortality was 41.7% in the total study population. Though initiation of oral beta-blockers during hospitalization was independently associated with a lower risk of in-hospital all-cause mortality, no benefit of initiation of oral beta-blockers was found on risk of 2-year all-cause mortality after adjusting for confounding factors, and it exhibited a trend toward increased 2-year all-cause mortality, especially in county hospitals. Upon using IPTW to balance the baseline covariates and further adjusting for discharge medication, initiation of oral beta-blockers was associated with increased risk of 2-year all-cause death. These results provide some important information and insights for the initiation of oral beta-blockers during hospitalization on long-term outcomes in STEMI patients with CS.

A report from the National Cardiovascular Data Registry (NCDR) showed an in-hospital mortality rate of 33.1% in patients with STEMI complicated by CS (28). Another report from the National Inpatient Sample database demonstrated that in-hospital mortality of STEMI patients with CS decreased significantly from 44.6% to 33.8% (29). More recently, Aissaoui et al. (8) used data from three nationwide French registries and reported that in-hospital mortality remained unchanged from 2005 to 2015 (41.8% to 37.8%) in AMI patients developing CS on admission. They also observed a decrease in 1-year mortality from 60% to 37.8% over the 10-year period (8). In addition to these registry data, several randomized clinical trials reported 30-day all-cause mortality ranging from 40% to 60% in AMI patients with CS (4, 23, 30, 31). In our analysis, the in-hospital, 30-day, and 1-year all-cause mortality were 33.2, 35.0, 39.8%, respectively, which were similar to the results of most previous studies.

Venkatason et al. (32) performed a retrospective analysis of 1,753 Malaysian STEMI patients from 2006 to 2013, and found that the administration rate of beta-blockers during hospitalization was 51.1%. More recently, Aissaoui et al. (8) used data from three nationwide French registries and reported that the percentage of patients receiving beta-blocker therapy during the first 48 h was 33, 35 and 43% in AMI patients with CS on admission in 2005, 2010, and 2015, respectively, with increasing rates of primary PCI over the same period. The latter one was close to the results of our study with patients enrolled from 2013 to 2014. A slight increase in in-hospital initiation of beta-blockers in recent years may be due to the popularization of early revascularization which facilitates hemodynamic stabilization, and that explains our finding that patients initiating oral beta-blockers were more likely to receive primary PCI. Elgendy et al. (33) used data from the NCDR Chest Pain-MI registry between 2008 and 2017, and showed that beta-blockers were administered in 55.6% of AMI patients presenting with CS within 24 h of admission. This rate was significantly higher in males than females (56.5% vs. 54.1%), which was consistent with our finding that more male patients were present in the group initiating oral beta-blockers. In the study of van Diepen et al. (20) in which 240 AMI patients with CS were included, they found that patients administered beta-blockers were more likely to receive aspirin, statin and diuretic therapy in the first 24 h. However, to date, few studies have systematically characterized the particular group who initiated in-hospital beta-blockers among STEMI patients with CS in real-world settings. Our study found that they were in better condition, more frequently to receive primary PCI and secondary prevention (Graphical abstract). Such patients were relatively hemodynamically more stable, and thus more likely to tolerate beta-blockers. Besides, in-hospital initiation of beta-blockers is more frequent in provincial hospitals, and this may be attributed to the increased understanding of its beneficial effect on the prognosis of AMI. However, are oral beta-blocker really beneficial for long-term mortality in these critically ill STEMI patients with CS?

van Diepen et al. (20) performed a secondary analysis of the TRIUMPH trial, which included 240 AMI patients with CS lasting more than 24 h. In their analysis, it was reported that patients administered beta-blockers within the first 24 h after CS diagnosis had higher 30-day mortality compared with those not receiving this early therapy (33.3% vs. 16.9%, *P* = 0.017) (20). Delmas et al. (34) retrospectively reviewed data of 275 patients with CS in a center in France from 2013 to 2014, in which the leading cause of CS was MI (35.3%). They found that previous use of beta-blockers was associated with a lower risk of long-term (≥ 2 years) mortality (HR = 0.61, 95% CI: 0.41–0.89, *P* = 0.02). However, there was no significant difference in long-term mortality between patients using beta-blockers at discharge or not (46.7% vs. 53.3%, *P* = 0.09). Di Santo et al. (19) investigated the impact of baseline beta-blockers on clinical outcomes in 192 CS patients based on the data of the DOREMI trial; there was no difference in the in-hospital all-cause mortality between patients treated with beta-blockers or not in the adjusted models (RR = 1.05, 95% CI: 0.70–1.58, *P* = 0.81). In the above-mentioned study of Aissaoui et al. (8) based on three nationwide French registries, use of beta-blockers within 48 h of admission was not correlated with 1-year mortality in AMI patients presenting with CS at admission (HR = 0.73, 95% CI: 0.52–1.02, *P* = 0.069). The reason for the conflicting findings might be attributed to the inconsistency in the timing of beta-blockers administration. For example, Delmas et al. (34) focused on the effect of baseline beta-blockers, which meant that beta-blockers were previously administered before the occurrence of CS, but the beneficial effect of beta-blockers was lost when considering beta-blockers at discharge. In our study, oral beta-blockers were initiated during hospitalization in STEMI patients with CS. Though patients with oral beta-blockers seemed to have a better in-hospital survival, no protective effect of oral beta-blockers was found on 30-day, 1-year and 2-year all-cause mortality in the fully-adjusted Cox regression model (Graphical abstract). Besides, there seemed to be a harmful effect of oral beta-blockers on 2-year all-cause mortality by using IPTW and adjusting for discharge medication. The negative inotropic effect of beta-blockers can cause hemodynamic deterioration and blunt vasopressor response in CS patients, thereby increasing long-term mortality or at least negating its benefits (20, 35).

According to the European Society of Cardiology (ESC) guidelines for the management of STEMI in 2017, intravenous beta-blockers should be avoided in patients with hypotension (10), but the timing of initiation of oral beta-blockers in patients with CS has not been clearly defined. In the recent guideline of American College of Cardiology Foundation/American Heart Association (ACCF/AHA), oral beta-blockers are not recommended for STEMI patients at high risk of CS or in low-output states (13, 18); the guidelines also suggest that patients with initial contraindications in the first 24 h after STEMI should undergo re-evaluation of their eligibility for initiating beta-blockers (13, 18). Although beta-blocker therapy has been shown to exert beneficial effects in critically ill patients (36, 37), our findings align with these guideline recommendations and confirm that initiating oral beta-blockers during hospitalization had no benefit on long-term mortality in STEMI patients with CS. In our study, patients who initiated oral beta-blockers were generally in significantly better condition than those who did not, which was consistent with the findings of Zhang et al. (38) from the China PEACE study. This difference was reflected not only in more favorable baseline indicators but also in these patients’ ability to undergo PCI and tolerate standardized secondary preventive treatment at discharge. Since oral beta-blockers in our study were all prescribed cautiously following comprehensive assessment by clinicians, tolerance to oral beta-blockers may serve as an important marker for evaluating disease severity and long-term prognosis. In subgroup and interaction analyses, we found that initiating oral beta-blockers was independently associated with higher long-term mortality in county hospitals. As county-level hospitals are located in the smallest cities in China and are typically small in scale, clinicians at these hospitals may lack sufficient clinical experience in managing CS, leading to suboptimal judgment regarding the timing of beta-blocker initiation and consequently increased mortality. These findings highlight that the initiation of oral beta-blockers during hospitalization requires extreme caution in STEMI patients with CS; otherwise, inappropriate initiation is not only ineffective but may even be harmful.

The present study had several limitations. First, the CAMI registry is an observational study, and our conclusions may thus be affected by confounding factors due to the non-randomized nature of our analysis. Though we performed multivariable regression and IPTW analysis to account for potential confounders, unmeasured confounders still exist and may limit inferences regarding causation. Theoretically, well-designed randomized clinical trials are needed in the future to further verify these results, but it may be challenging to conduct randomized clinical trials in such critically ill patients. Second, the absence of systematic hemodynamic monitoring precluded the use of more refined shock classification systems, such as the SCAI criteria. Third, despite the multicenter design of our study, all included patients were Chinese, so extrapolation of our conclusions to other ethnic groups should be done with caution. Fourth, the exact timing of in-hospital beta-blocker initiation for each patient was not accurately documented. However, in clinical practice, oral beta-blockers for patients with CS are typically initiated after clinicians carefully weigh the risks and benefits, usually once hemodynamic stability is achieved. Even so, we still found no protective effect of in-hospital oral beta-blocker initiation on long-term mortality. Finally, the doses, titration strategies, and types of in-hospital oral beta-blockers, as well as patients’ long-term adherence were not specifically documented, which may affect outcomes. Therefore, further studies are warranted to investigate how different doses and types of beta-blockers administered during hospitalization and during follow-up affect the long-term prognosis of STEMI patients with CS.

## 5 Conclusion

In conclusion, nearly half of Chinese patients with CS complicating STEMI initiated oral beta-blocker therapy during hospitalization. Patients who received oral beta-blockers were in better condition, and more likely to receive primary PCI and secondary prevention at discharge. The 2-year all-cause mortality was 41.7% and the majority of deaths occurred during hospitalization. No benefit of initiating oral beta-blockers was found on long-term all-cause mortality after adjusting for confounding factors, and a trend toward increased mortality existed, especially in small-scale hospitals with insufficient experience in CS treatment.

## Data Availability

The raw data supporting the conclusions of this article will be made available by the authors, without undue reservation.
