# Supplementary material for: Impact of in-hospital oral beta-blockers initiation on long-term outcomes in ST-elevation myocardial infarction patients with cardiogenic shock
Source: Front Med (Lausanne). 2025 Oct 13;12:1666977. doi: 10.3389/fmed.2025.1666977 (PMC12554648; doi:10.3389/fmed.2025.1666977)
Supplement: Supplementary file 1 [file Data_Sheet_1.docx]

**Supplementary Table S1.** **Assessment of multicollinearity using VIF in the multivariable Cox regression model**

| Variable | VIF |
| --- | --- |
| Sex (Male/Female) | 1.12 |
| Age (<75 / ≥75 years) | 1.11 |
| History of diabetes (Yes/No) | 1.04 |
| History of hypertension (Yes/No) | 1.07 |
| History of HF (Yes/No) | 1.01 |
| Heart rate (≤100 / >100 bpm) | 1.10 |
| Infarct location (Anterior/Non-anterior) | 1.09 |
| Primary PCI (Yes/No) | 1.17 |
| Discharge medication^*^ |  |
| Aspirin (Yes/No) | 6.94 |
| P2Y12 receptor inhibitor (Yes/No) | 5.84 |
| Statin (Yes/No) | 5.11 |
| Level of hospital |  |
| Prefectural | 1.74 |
| Provincial | 1.87 |

VIF, variance inflation factor; HF, heart failure; PCI, percutaneous coronary intervention.

A VIF value < 5 indicates low multicollinearity, a value ranging from 5 to 10 indicates moderate multicollinearity, and a value ≥ 10 indicates high multicollinearity.

^*^The moderate VIF values for the discharge medications are clinically expected as part of standard guideline-directed therapy.

### Supplemental Table S2. SMD of the variables included in the inverse propensity score weighting before and after adjustment

|  | With beta-blockers  (n=299) | Without beta-blockers  (n=401) | SMD before IPTW | SMD after IPTW |
| --- | --- | --- | --- | --- |
| Age≥75 years, n (%) | 70(23.4) | 97(24.2) | 0.334 | 0.019 |
| Male, n (%) | 212(70.9) | 282(70.5) | 0.328 | 0.010 |
| Current smoking, n (%) | 117(39.2) | 156(38.9) | 0.203 | 0.006 |
| Level of hospital, n (%) |  |  |  |  |
| provincial | 76(25.5) | 100(24.9) | 0.199 | 0.014 |
| prefectural | 160(53.5) | 218(54.3) | 0.000 | 0.016 |
| county | 63(21.0) | 83(20.8) | 0.211 | 0.004 |
| History of hypertension, n (%) | 135(45.0) | 182(45.3) | 0.032 | 0.007 |
| History of diabetes, n (%) | 51(16.9) | 68(17.1) | 0.113 | 0.005 |
| Prior HF, n (%) | 10(3.3) | 12(3.0) | 0.136 | 0.022 |
| HR>100 beats/min, n (%) | 65(21.6) | 87(21.6) | 0.110 | 0.001 |
| SBP, mean ± SD, mmHg | 92.3 ±26.5 | 91.9 ±27.6 | 0.237 | 0.016 |
| GRACE Score, mean ± SD | 166.5 ±28.4 | 166.9±31.5 | 0.268 | 0.001 |
| Anterior MI, n (%) | 131(43.7) | 177(44.2) | 0.091 | 0.009 |
| Primary PCI, n (%) | 110(36.7) | 144(36.0) | 0.250 | 0.013 |

Abbreviations: HF, heart failure; HR, heart rate; MI, myocardial infarction; PCI, percutaneous coronary intervention; SBP, systolic blood pressure; SD, standard deviation；SMD, standardized mean differences

### Supplemental Table S3. Discharge medications of study population stratified by initiation of oral beta-blockers.

|  | Total ^a^  (n=497) | With beta-blockers  (n=265) | Without beta-blockers  (n=232) | *P* value |
| --- | --- | --- | --- | --- |
| Aspirin, n (%) | 428 (86.1) | 256(96.6) | 172(74.1) | <0.001 |
| P2Y12 receptor inhibitor, n (%) | 417 (83.9) | 248(93.6) | 169(72.8) | <0.001 |
| Statin, n (%) | 416 (83.7) | 250(94.3) | 166(71.6) | <0.001 |
| Calcium antagonist, n (%) | 22 (4.4) | 16(6.0) | 6(2.6) | 0.057 |
| ACEI/ARB, n (%) | 213 (42.9) | 163(61.5) | 50(21.6) | <0.001 |
| MRA, n (%) | 186 (37.4) | 104(39.2) | 82 (35.3) | 0.370 |
| Diuretics, n (%) | 143 (28.8) | 84(31.7) | 59 (25.4) | 0.123 |
| Nitrates, n (%) | 227 (45.7) | 152(57.4) | 75 (32.3) | <0.001 |

^a^ Only subjects who are alive at discharge were taken into account.

Abbreviations: ACEI/ARB, angiotensin-converting enzyme inhibitor/angiotensin receptor blocker; MRA, mineral-corticoid receptor antagonist.

### Supplemental Table S4. Impact of oral beta-blockers on outcomes in the whole population in Cox regression model.

| Outcome | Crude model | | Fully adjusted model^b^ | |
| --- | --- | --- | --- | --- |
|  | HR (95% CI) | *P* value | HR (95% CI) | *P* value |
| **In-hospital** |  |  |  |  |
| All-cause death | 0.31 (0.23, 0.42) | <0.001 | 0.37 (0.27, 0.50) | <0.001 |
| MACCE ^a^ | 0.34 (0.26, 0.45) | <0.001 | 0.40 (0.29, 0.53) | <0.001 |
| Recurrent MI | 0.72 (0.23, 2.28) | 0.572 | 0.62 (0.19, 2.06) | 0.435 |
| Stroke | 0.63 (0.23, 1.72) | 0.369 | 0.79 (0.27, 2.26) | 0.655 |
| TIMI major bleeding | 0.27 (0.05, 1.40) | 0.118 | 0.24 (0.04, 1.42) | 0.116 |
| **30-day follow-up** |  |  |  |  |
| All-cause death | 0.31 (0.23, 0.42) | <0.001 | 1.36 (0.98, 1.90) | 0.068 |
| MACCE ^a^ | 0.34 (0.26, 0.45) | <0.001 | 1.39 (1.01, 1.90) | 0.045 |
| Recurrent MI | 0.62 (0.20, 1.91) | 0.403 | 4.10 (1.02, 16.40) | 0.046 |
| Stroke | 0.73 (0.28, 1.92) | 0.527 | 1.44 (0.42, 4.90) | 0.558 |
| Revascularization | 0.79 (0.36, 1.70) | 0.542 | 0.96 (0.41, 2.26) | 0.920 |
| TIMI major bleeding | 0.27 (0.05, 1.40) | 0.117 | 0.37 (0.04, 3.24) | 0.372 |
| **1-year follow-up** |  |  |  |  |
| All-cause death | 0.34 (0.26, 0.44) | <0.001 | 1.26 (0.92, 1.73) | 0.143 |
| MACCE ^a^ | 0.37 (0.28, 0.47) | <0.001 | 1.29 (0.95, 1.74) | 0.099 |
| Recurrent MI | 0.85 (0.33, 2.20) | 0.732 | 3.26 (0.93, 11.37) | 0.064 |
| Stroke | 0.60 (0.24, 1.49) | 0.269 | 1.08 (0.33, 3.55) | 0.897 |
| Revascularization | 0.88 (0.47, 1.64) | 0.684 | 1.09 (0.54, 2.17) | 0.817 |
| TIMI major bleeding | 0.41 (0.10, 1.76) | 0.233 | 0.56 (0.08, 4.00) | 0.567 |
| **2-year follow-up** |  |  |  |  |
| All-cause death | 0.35 (0.27, 0.46) | <0.001 | 1.29 (0.95, 1.75) | 0.099 |
| MACCE ^a^ | 0.37 (0.29, 0.48) | <0.001 | 1.26 (0.94, 1.68) | 0.126 |
| Recurrent MI | 0.68 (0.27, 1.69) | 0.406 | 1.93 (0.59, 6.35) | 0.280 |
| Stroke | 0.54 (0.22, 1.34) | 0.184 | 0.87 (0.28, 2.71) | 0.812 |
| Revascularization | 0.70 (0.40, 1.23) | 0.216 | 0.81 (0.44, 1.50) | 0.501 |
| TIMI major bleeding | 0.56 (0.15, 2.14) | 0.401 | 0.87 (0.15, 5.13) | 0.873 |

^a^ MACCE represents a composite of all-cause death, recurrent MI and stroke.

^b^ For in-hospital outcomes, age, gender, history of diabetes, hypertension or HF, heart rate, anterior MI, primary PCI and level of hospital were adjusted. For outcomes at follow-up, age, gender, history of diabetes, hypertension or HF, heart rate, anterior MI, primary PCI, level of hospital and discharge medication including aspirin, P2Y12 receptor inhibitor and statin were adjusted (equals to adjusted model 3).

Abbreviations: CI, confidence interval; HF, heart failure; MACCE, major adverse cardiovascular and cerebrovascular event; MI, myocardial infarction; OR, odds ratio.

### Supplemental Table S5. Landmark analysis discriminating effects of oral beta-blockers on all-cause mortality before and after 30 days of follow-up

|  | Crude model | | Adjusted model 1 | | Adjusted model 2 | | Adjusted model 3 | |
| --- | --- | --- | --- | --- | --- | --- | --- | --- |
|  | HR (95%CI) | *P* value | HR (95%CI) | *P* value | HR (95%CI) | *P* value | HR (95%CI) | *P* value |
| 30-day | 0.31(0.23,0.42) | <0.001 | 0.34(0.25,0.46) | <0.001 | 0.37(0.27,0.50) | <0.001 | 1.36(0.98,1.90) | 0.068 |
| >30-day–2 years ^a^ | 0.64(0.35,1.16) | 0.139 | 0.76(0.42,1.39) | 0.377 | 0.83(0.45,1.52) | 0.537 | 0.98(0.50,1.93) | 0.958 |

Variables included in the adjusted Cox regression model 1 were: initiation of oral beta-blockers, age, gender.

Variables included in the adjusted Cox regression model 2 were: initiation of oral beta-blockers, age, gender, primary PCI.

Variables included in the adjusted Cox regression model 3 (fully adjusted model) were: initiation of oral beta-blockers, age, gender, history of diabetes, hypertension or HF, heart rate, anterior MI, primary PCI, level of hospital and discharge medication including aspirin, P2Y12 receptor inhibitor and statin.

^a^ Landmark analysis after exclusion of patients died within 30 days.

Abbreviations: CI, confidence interval; HF, heart failure; MI, myocardial infarction; HR, hazard ratio; PCI, percutaneous coronary intervention.

### Supplemental Table S6. Baseline characteristics and treatment during hospitalization of study population stratified by level of hospital

|  | Provincial hospital | | | Prefectural hospital | | | County hospital | | | |
| --- | --- | --- | --- | --- | --- | --- | --- | --- | --- | --- |
|  | With beta-blockers（N=93） | Without beta-blockers  （N=88） | P value | With beta-blockers（N=171） | Without beta-blockers  （N=229） | P value | With beta-blockers（N=54） | Without beta-blockers  （N=109） | P value | |
| Age, mean ± SD, years | 61.2±11.5 | 62.8±12.0 | 0.374 | 61.9±12.0 | 67.7±11.7 | <0.001 | 67.5±11.9 | 68.9±12.8 | 0.511 | |
| Age≥75 years, n (%) | 10(10.8) | 12(13.6) | 0.553 | 27(15.8) | 75(32.8) | <0.001 | 15(27.8) | 42(38.5) | 0.171 | |
| Male, n (%) | 75(80.6) | 67(76.1) | 0.461 | 137(80.1) | 148(64.6) | <0.001 | 39(72.2) | 59(54.1) | 0.024 | |
| BMI, mean ± SD, kg/m^2^ | 25.8±15.2 | 24.0±2.6 | 0.293 | 23.6±2.8 | 23.7±3.0 | 0.656 | 22.7±3.2 | 23.5±2.8 | 0.084 | |
| Current smoking, n (%) | 46(49.5) | 38(43.7) | 0.437 | 78(45.6) | 75(32.8) | 0.009 | 18(33.3) | 34(31.8) | 0.842 | |
| History of disease |  |  |  |  |  |  |  |  |  | |
| Hypertension, n (%) | 43(46.2) | 47(53.4) | 0.335 | 77(45.0) | 95(41.5) | 0.479 | 22(40.7) | 55(50.5) | 0.241 | |
| Diabetes, n (%) | 18(19.4) | 17(19.3) | 0.995 | 23(13.5) | 47(20.5) | 0.063 | 6(11.1) | 17(15.6) | 0.431 | |
| Hyperlipidemia, n (%) | 8(10.5) | 9(12.0) | 0.775 | 10(6.7) | 2(1.1) | 0.007 | 2(5.3) | 4(5.6) | 1.000^a^ | |
| Prior MI, n (%) | 3(3.6) | 10(12.8) | 0.027 | 13(7.7) | 14(6.7) | 0.698 | 1(2.1) | 5(5.1) | 0.664^a^ | |
| Prior PCI, n (%) | 6(7.1) | 8(9.6) | 0.545 | 9(5.3) | 10(4.4) | 0.680 | 0(0.0) | 3(3.0) | 0.552^a^ | |
| Prior CABG, n (%) | 0(0) | 0(0) | - | 0(0.0) | 1(0.4) | 1.000^a^ | 0(0.0) | 0(0.0) | - | |
| Prior HF, n (%) | 2(2.2) | 3(3.4) | 0.676^a^ | 1(0.6) | 11(4.8) | 0.007 | 2(3.7) | 2(1.8) | 0.600^a^ | |
| Prior non-hemorrhagic stroke, n (%) | 7(7.7) | 11(13.3) | 0.228 | 13(7.6) | 28(12.4) | 0.112 | 5(9.6) | 5(4.9) | 0.305^a^ | |
| On admission |  |  |  |  |  |  |  |  |  | |
| Physical and auxiliary examination |  |  |  |  |  |  |  |  |  | |
| HR, median (IQR), beats/min | 80.0(62.0, 96.0） | 74.0(60.0,99.5) | 0.796 | 71.0(52.0,92.0) | 78.0(54.0 ,100.0) | 0.085 | 73.5(56.0,107.0) | 60.0(44.0,92.0) | 0.090 | |
| HR>100 beats/min, n (%) | 18(19.4) | 22(25.0) | 0.360 | 29(17.0) | 56(24.5) | 0.068 | 14(25.9) | 23(21.1) | 0.492 | |
| SBP, mean ± SD, mmHg | 98.9±26.9 | 93.0±22.9 | 0.121 | 97.2±28.4 | 90.0±28.5 | 0.015 | 86.5±23.5 | 84.8±30.1 | 0.716 | |
| LVEF, % | 50.4±12.1 | 48.5±11.9 | 0.348 | 50.6±11.9 | 51.1±13.9 | 0.784 | 53.9±9.8 | 48.5±12.2 | 0.070 | |
| Hemoglobin, mean ± SD, g/L | 138.7±20.7 | 133.8±23.0 | 0.134 | 134.5±19.5 | 129.2±25.7 | 0.024 | 128.1±21.7 | 132.5±23.3 | 0.273 |  |
| Scr, median (IQR), mg/dL | 80.8(67.2, 102.0) | 93.9(73.7, 138.5) | 0.187 | 95.0(77.0, 123.0) | 96.3(71.0, 131.0) | 0.323 | 89.0(65.6, 111.0) | 99.1(76.8, 121.0) | 0.108 |  |
| Other characteristics |  |  |  |  |  |  |  |  |  |  |
| GRACE Score, mean ± SD | 155.2 ±30.3 | 162.8 ±32.8 | 0.107 | 157.8±29.2 | 170.3±30.7 | <0.001 | 173.7±31.6 | 168.2±35.3 | 0.330 |  |
| Anterior MI, n (%) | 50(53.8) | 40(45.5) | 0.264 | 80(46.8) | 101(44.1) | 0.594 | 21(38.9) | 42(38.5) | 0.965 |  |
| Right ventricular MI, n (%) | 21(23.1) | 22(25.0) | 0.763 | 32(18.7) | 45(19.7) | 0.814 | 9(16.7) | 26(23.9) | 0.285 |  |
| Use of vasoactive agents, n (%) | 29(31.2) | 45(51.1) | 0.006 | 32(18.7) | 49(21.4) | 0.508 | 12(22.2) | 35(32.1) | 0.183 |  |
| Pre-hospital delay, n (%) |  |  | 0.865 |  |  | 0.825 |  |  | 0.736 |  |
| <3h | 21(23.1) | 17(19.3) |  | 54(32.0) | 68(30.0) |  | 23(42.6) | 49(45.4) |  |  |
| 3-6h | 24(26.4) | 22(25.0) |  | 54(32.0) | 67(29.5) |  | 13(24.1) | 25(23.1) |  |  |
| 6-12h | 15(16.5) | 14(15.9) |  | 20(11.8) | 28(12.3) |  | 8(14.8) | 10(9.3) |  |  |
| ≥12h | 31(34.1) | 35(39.8) |  | 41(24.3) | 64(28.2) |  | 10(18.5) | 24(22.2) |  |  |
| Treatment strategy |  |  |  |  |  |  |  |  |  |  |
| Primary PCI, n (%) | 55(59.1) | 39(44.3) | 0.046 | 73(42.7) | 81(35.4) | 0.137 | 7(13.0) | 10(9.2) | 0.463 |  |
| Thrombolysis, n (%) | 5(5.4) | 5(5.7) | 1.000^a^ | 24(14.0) | 27(11.8) | 0.507 | 23(42.6) | 33(30.3) | 0.122 |  |
| Emergency CABG, n (%) | 0 (0) | 0(0) | - | 1(0.6) | 1(0.4) | 1.000^a^ | 1(1.9) | 0(0.0) | 0.331^a^ |  |
| Adjunctive therapy |  |  |  |  |  |  |  |  |  |  |
| Temporary pacemaker, n (%) | 9(9.7) | 10(11.4) | 0.712 | 13(7.6) | 21(9.2) | 0.576 | 2(3.7) | 9(8.3) | 0.341^a^ |  |
| IABP, n (%) | 23(25.0) | 32(36.8) | 0.087 | 16(9.4) | 24(10.6) | 0.678 | 1(1.9) | 4(3.7) | 1.000^a^ |  |
| In-hospital complications |  |  |  |  |  |  |  |  |  |  |
| Mechanical complications, n (%) | 1(1.1) | 3(3.4) | 0.360^a^ | 3(1.8) | 6(2.6) | 0.738^a^ | 0(0.0) | 5(4.6) | 0.174^a^ |  |
| Cardiac arrest, n (%) | 8(8.7) | 17(19.3) | 0.038 | 19(11.2) | 84(37.0) | <0.001 | 10(18.9) | 36(33.0) | 0.055 |  |
| VT/VF, n (%) | 15(16.1) | 20(22.7) | 0.261 | 36(21.1) | 58(25.6) | 0.294 | 8(15.1) | 27(24.8) | 0.151 |  |
| Atrial flutter/atrial fibrillation, n (%) | 1(1.1) | 7(8.0) | 0.031^a^ | 11(6.4) | 18(7.9) | 0.568 | 2(3.8) | 11(10.1) | 0.224^a^ |  |
| Sinus arrest/severe bradycardia, n (%) | 0(0.0) | 2(2.3) | 0.235^a^ | 7(4.1) | 25(11.0) | 0.009 | 2(3.8) | 13(11.9) | 0.147^a^ |  |
| Second degree AVB or above, n (%) | 10(10.8) | 10(11.4) | 0.896 | 16(9.4) | 27(11.9) | 0.417 | 5(9.4) | 21(19.3) | 0.096 |  |
| Length of stay |  |  |  |  |  |  |  |  |  |  |
| Average length of stay, median (IQR), day | 11.0(8.0，16.0） | 9.0(3.0,13.0) | 0.042 | 13.0(8.0, 17.0) | 5.0(1.0,12.5) | <0.001 | 9.5(2.0,15.0) | 3.0(1.0, 11.0) | 0.003 |  |
| In the intensive care unit, median (IQR), day | 6.5(3.0, 9.5) | 4.0(1.0, 7.0) | 0.031 | 3.0(0.0,8.0) | 1.0(0.0,4.0) | <0.001 | 2.0(1.0,6.0) | 1.0(1.0,4.0) | 0.513 |  |
| In the general ward, median (IQR), day | 5.0(1.0, 8.0) | 2.0(0.0, 8.0) | 0.392 | 7.0(1.0,13.0) | 1.0(0.0,9.0) | <0.001 | 5.0(0.0,10.0) | 0.0(0.0,5.0) | 0.001 |  |
| Transfer to superior hospital | 0 (0) | 0(0) | - | 6(3.9) | 10(6.2) | 0.340 | 5(11.4) | 23(30.7) | 0.013 |  |

Abbreviations: BMI, body mass index; CABG, coronary artery bypass graft; HF, heart failure; HR, heart rate; IABP, intra-aortic balloon pump; IQR, interquartile range; LVEF, left ventricular ejection fraction; MI, myocardial infarction; PCI, percutaneous coronary intervention; SBP, systolic blood pressure; Scr, serum creatinine; SD, standard deviation.

^a^ Fisher’s exact test was used.

### Supplemental Figure S1. Kaplan–Meier curves for all-cause mortality in fully adjusted model ^a^. (A) All-cause mortality at 30 days. (B) Landmark analysis discriminating between all-cause death occurring before and after 30 days of follow-up

^a^ Adjusted for age, gender, history of diabetes, hypertension or heart failure, heart rate, anterior MI, primary PCI, level of hospital and discharge medication including aspirin, P2Y12 receptor inhibitor and statin.

Abbreviations: MI, myocardial infarction; PCI, percutaneous coronary intervention.

### Supplemental Figure S2. Kaplan–Meier curves for all-cause mortality in the subgroup of three level hospitals ^a^. (A)In the subgroup of

**provincial hospital. (B) In the subgroup of prefectural hospital. (C) In the subgroup of county hospital.**

^a^ Adjusted for age, gender, history of diabetes, hypertension or heart failure, heart rate, anterior MI, primary PCI, and discharge medication including aspirin, P2Y12 receptor inhibitor and statin.

Abbreviations: MI, myocardial infarction; PCI, percutaneous coronary intervention.
